# Management of Antithrombotic Therapy in Patients With Coexisting Atrial Fibrillation and Coronary Artery Disease Who Underwent Percutaneous Coronary Intervention Within the Last Year

**DOI:** 10.1002/clc.70196

**Published:** 2025-10-16

**Authors:** Sebastian König, Sven Hohenstein, Johannes Leiner, Anne Nitsche, Alexander Staudt, Frank Steinborn, Christian Bietau, Henning T. Baberg, Michael Niehaus, Jürgen Tebbenjohanns, Melchior Seyfarth, Markus W. Ferrari, Marc M. Vorpahl, Ralf Kuhlen, Kerstin Bode, Andreas Bollmann

**Affiliations:** ^1^ Department of Electrophysiology Heart Center Leipzig at University of Leipzig Leipzig Germany; ^2^ Helios Health Institute Clinical Trial Management & Real‐World Data Berlin Germany; ^3^ Department of Cardiology and Angiology Helios Hospital Schwerin Schwerin Germany; ^4^ Department of Cardiology/Interventional Electrophysiology Helios Hospital Erfurt Erfurt Germany; ^5^ Department of Cardiology and Angiology Helios Hospital Plauen Plauen Germany; ^6^ Department of Cardiology and Nephrology Helios Hospital Berlin‐Buch Berlin Germany; ^7^ Department of Internal Medicine I Helios Hospital Gifhorn Gifhorn Germany; ^8^ Department of Cardiology Helios Hospital Hildesheim Hildesheim Germany; ^9^ Department of Cardiology Helios University Hospital Wuppertal Wuppertal Germany; ^10^ Helios Dr. Horst Schmidt Kliniken Wiesbaden Clinic of Internal Medicine 1 Wiesbaden Germany; ^11^ Department of Cardiology and Angiology Helios Hospital Siegburg Siegburg Germany; ^12^ Fresenius Medical Care AG Bad Homburg v.d. Höhe Germany

**Keywords:** anticoagulation, antithrombotic treatment, atrial fibrillation, coronary artery disease, percutaneous coronary intervention

## Abstract

**Background:**

Coronary artery disease (CAD) is a common comorbidity in patients with atrial fibrillation (AF), and optimal antithrombotic medication improves clinical outcomes in this high‐risk population. The aim of our study was to describe antithrombotic drug regimens in different patient cohorts.

**Methods:**

We investigated data from the prospective Helios Heart registry (H2) and the Heart Center Leipzig routine clinical database (HZL). We included inpatient cases with AF and CAD (hospitalized from March 2021 to July 2024 [H2] or January 2017 to December 2021 [HZL]), who underwent percutaneous coronary intervention (PCI) within the last 12 months. Information on clinical characteristics, coronary interventions, and medication prescribed was obtained from electronic case report forms and/or administrative data based on ICD‐10, OPS, and ATC codes.

**Results:**

We included 3481 (HZL), and 205 (H2) index cases with comparable baseline characteristics. Overall, 92.5% (HZL) and 87.6% (H2) of patients were on any anticoagulation, and 93.0% (HZL) and 80.2% (H2) were prescribed ≥ 1 antiplatelet agent. There were relevant differences in antithrombotic therapy when stratifying for PCI timing. Factors associated with higher (older age) or lower (comorbidity burden, antiplatelet treatment, prior left atrial appendage occlusion) prescription rates of OAC were identified. OAC therapy without adjunctive antiplatelet therapy was associated with an increased rate of rehospitalization for major adverse cardiovascular events at 12 months (HZL).

**Conclusion:**

We presented current data on antithrombotic drug utilization in patients with AF and CAD and found comorbidity burden, concomitant antiplatelet treatment and other factors to be associated with lower anticoagulant prescription rates.

## Graphical abstract

This study analyzed two high‐quality clinical databases comprising 205 and 3481 patients with atrial fibrillation and coronary artery disease who underwent percutaneous coronary intervention within the last 12 months. We described treatment patterns of antithrombotic drug therapy and identified factors that were associated with such medication.

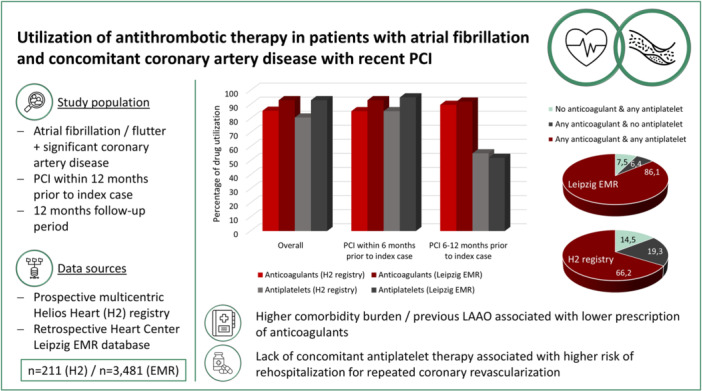

**Abbreviations**
AF: Atrial fibrillation
ATC: Anatomical therapeutic chemical
CAD: Coronary artery disease
CI: Confidence interval
DAPT: Dual antiplatelet therapy
EMR: Electronic medical records
ePCI: Earlier percutaneous coronary intervention
EORP‐AF: EurObservational Research Programme in Atrial Fibrillation
FU: Follow‐up
H2 registry: Helios Heart registry
ICD‐10: International Statistical Classification of Diseases and Related Health Problems
LAAO: Left atrial appendage occlusion
LVEF: Left ventricular ejection fraction
MACE: Major adverse cardiovascular events
NOAC: Non‐vitamin K antagonist oral anticoagulation
OAC: Oral anticoagulant
OPS: Operations and Procedures (German adaptation of the International Classification of the Procedures in Medicine of the World Health Organization, version 2017)
OR: Odds ratio
PCI: Percutaneous coronary intervention
rPCI: Recent percutaneous coronary intervention
VKA: Vitamin K antagonist

## 1 Introduction

Atrial fibrillation (AF) is the most common cardiac arrhythmia in adults with a significant impact on morbidity, mortality, and overall healthcare utilization [1, 2, 3, 4]. As the number of patients with AF is expected to increase, there is a need to continuously optimize their treatment in accordance to guidelines and the available scientific data [5, 6, 7]. This includes oral anticoagulant (OAC) therapy for patients with a corresponding risk profile, which has been shown to reduce rates of stroke, systemic embolism, and mortality [6, 8, 9]. Coronary artery disease (CAD) is a risk modifier and a common comorbidity in patients with AF, with a reported prevalence of up to 40% [10, 11, 12, 13, 14]. The coexistence of AF and CAD has mutual influence on higher rates of adverse events and challenges physicians in providing antithrombotic treatment that balances ischemic and bleeding risks [15, 16, 17, 18]. The optimal antithrombotic treatment strategy has been the subject of several large clinical trials, leading to differentiated recommendations in current European guidelines with regard to the indication and duration of a combination of anticoagulants and antiplatelets [7, 19, 20, 21, 22, 23]. However, there is a paucity of data on the real‐world utilization of antithrombotic therapy in patients with coexisting AF and CAD who have recently undergone percutaneous coronary intervention (PCI). Therefore, the aims of this study were to describe the prescription of different antithrombotic drug regimens, to characterize treatment patterns according to patients’ baseline characteristics, to compare antithrombotic drug therapy in different German inpatient datasets, and to investigate the impact of drug regimens on event rates during follow‐up (FU).

## 2 Methods

### 2.1 Data Sources

For the analyses underlying this study, we used two different data sources. First, we evaluated data of the prospective, non‐interventional, multicenter Helios Heart registry (H2 registry). The H2 registry has been enrolling inpatients with diagnosed heart failure (regardless of the reason for hospitalization or heart failure subtype and etiology) since March 2021 and is continuously recruiting in 10 German Helios hospitals with specialized cardiology departments. Patients with a study enrollment date through the end of July 2024 were eligible for this study. The prospective data assessment included patient interviews, the assessment of patient‐reported outcome measures, and the evaluation of electronic medical records (EMR) with a variable set being based on a standardized patient‐centered outcome measure according to the International Consortium for Health Outcomes Measurement [24]. Regular FUs are carried out every 6 months. The second dataset was derived from the EMR database of the Heart Center Leipzig, which includes digitally captured routine data from all inpatients treated in this tertiary center. It consists of both clinical information (vital signs, lab values, medication, imaging) and administrative data, the latter including main and secondary diagnoses at hospital discharge based on the International Statistical Classification of Diseases and Related Health Problems (ICD‐10 [German Modification]) encoding system, as well as performed procedures according to the encoded Operations and Procedures codes (OPS [German adaptation of the International Classification of the Procedures in Medicine of the World Health Organization, version 2017]).

Data of cases with an admission date between the 1st of January 2017 and the 31st of December 2021 were included. There was no imputing for missing data. Data was stored in a double‐pseudonymized form and data use was approved by the local ethics committee of the University of Leipzig and the Helios Kliniken GmbH data protection authority. Patients included in the H2 registry provided written informed consent. Considering the retrospective analysis of double‐pseudonymized administrative and EMR clinical routine data, individual informed consent was not obtained for the analysis of the Heart Center Leipzig EMR database. The study follows the STROBE guidelines for the reporting of observational studies.

### 2.2 Study Population

For cross‐sectional analyses, we included inpatient cases with diagnosed AF and concomitant significant CAD (epicardial stenosis ≥ 50% of at least one main coronary artery) with recent PCI (performed within 12 months before index case). For the Heart Center Leipzig EMR database, inclusion criteria were based on ICD‐10 (index case) and OPS codes (index case and/or previous inpatient cases). Detailed information on used ICD‐10 and OPS codes is provided in the Supporting Information S1: Table 1. For longitudinal analyses, the first case within the study period that met the eligibility criteria was considered the index case for all data sources. All following inpatient readmission within a 365‐day period from index hospital discharge were analyzed for FU readmission statistics (limited to readmissions to the hospital of the index hospital stay).

### 2.3 Variables of Interest

For the H2 registry cohort, information on comorbidities, performed medical procedures, and medication were derived from patient interviews or directly extracted from medical records. Since the primary focus of the H2 registry was not investigating antithrombotic drug treatment, its data did not differentiate between single or dual antiplatelet treatment. For the EMR dataset, comorbidities were identified via ICD‐10‐encoded information within administrative data and structured based on a modified Elixhauser comorbidity index (Supporting Information S1: Table 2) using the R comorbidity package [25, 26]. The CHA_2_DS_2_‐VASc score was calculated directly (H2 registry) or via ICD‐10‐encoded information (Supporting Information S1: Table 3). In‐hospital treatments of interest and variables relevant to outcome definition are listed in the Supplemental material (Supporting Information S1: Table 4). Data on medication was extracted using the Anatomical therapeutic chemical (ATC) encoding system (Supporting Information S1: Table 5). Information on left ventricular ejection fraction (LVEF) was derived from EMR data taking into account imaging studies that were performed up to 365 days before to 7 days after index hospital admission (preferring values closest to the study entry). LVEF data were structured according to existing imaging guidelines [27].

### 2.4 Statistics

Inferential statistics were based on the R environment for statistical computing (version 4.0.2, 64‐bit build) [28]. For all tests, we applied a two‐tailed 5% error criterion for significance. For the description of the patient characteristics and comorbidities, we employed χ2‐tests for categorical variables, and analysis of variance for numerical variables. We report proportions, means, standard deviations, and p values. For the comparison of proportions of selected treatments and outcomes in the different cohorts, we used logistic regression for dichotomous variables. We report proportions, odds ratios (OR) together with confidence intervals (CI) and p values. Categorical variables with more than two levels were analyzed with χ2‐tests. For multivariable analyses, categorical variables entered the analysis as simple‐coding contrasts while the continuous variable “age” was centered at its mean. All continuous variables were scaled to unit variance. The variable selection for the multivariate analysis was based on content considerations. A subgroup analysis compared patients with more recent PCI (rPCI, PCI within index case or up to 6 months before study entry) and PCI being performed between 6 and 12 months before patient's index case (earlier PCI or ePCI).

## 3 Results

### 3.1 H2 Registry

Of 2805 patients included into the H2 registry until the 31st of July 2024, 205 (7.3%) patients met the inclusion criteria for this study (mean age 74.6 ± 9.0 years, 30.7% female). The most common comorbidities were hypertension, chronic kidney disease, and type 2 diabetes mellitus, resulting in a mean CHA_2_DS_2_‐VASc score of 5.4 ± 1.4 (Table 1). A majority of patients (*n* = 178, 86.8%) underwent PCI within 6 months before or during index hospitalization (*n* = 134 or 65.4% of all patients with PCI during index hospitalization). A left atrial appendage occlusion (LAAO, interventional or surgical) performed during or before index hospitalization was reported in 4.4% of patients (*n* = 9). Baseline characteristics are detailed in Table 1.

**TABLE 1:** Baseline characteristics of index cases differentiated for data sources.

Variable	H2 registry^a^	Heart Center Leipzig EMR^a^
	n = 205	n = 3481
Mean age [years]	74.6 ± 9.0	75.8 ± 9.1
Sex, female [n (%)]	63 (30.7)	967 (27.8)
BMI [kg/m²]	28.5 ± 5.5	28.4 ± 5.1
CHA_2_DS_2_‐VASc score	5.4 ± 1.4	5.0 ± 1.3
Elixhauser comorbidity score	/	15.1 ± 12.1
LVEF^b^ [%]	41.8 ± 13.7	46.0 ± 14.5
eGFR^c^ [mL/min/1.73 m²]	53.0 ± 23.4	57.2 ± 23.2
PCI within index case or up to 6 months before study entry [n (%)]	178 (86.8)	3246 (93.2)
Hypertension [n (%)]	189 (92.2)	3151 (90.5)
Heart failure [n (%)]	211 (100.0)	2563 (73.6)
HFrEF based on LVEF [n (%)]	80 (44.0)^d^	1060 (36.1)^d^
CKD [n (%)]	122 (59.5)	1959 (56.3)
Diabetes mellitus [n (%)]	96 (46.8)	1558 (44.8)
PVD [n (%)]	66 (32.2)	1498 (43.0)
Cerebrovascular disease [n (%)]	35 (17.1)	144 (4.1)
Chronic pulmonary disease [n (%)]	42 (20.5)	336 (9.7)
In‐hospital mortality at index case [n (%)]	3 (1.5)	198 (6.6)^e^

Abbreviations: BMI, Body mass index; CKD, Chronic kidney disease; eGFR, Estimated glomerular filtration rate; EMR, Electronic medical records; HFrEF, Heart failure with reduced ejection fraction; PVD, Peripheral vascular disease.

^a^ Presented as means with standard deviation or proportions.

^b^ Missing data for *n* = 31 (H2 registry) and *n* = 616 (Leipzig EMR).

^c^ Missing data for *n* = 19 (H2 registry) and *n* = 241 (Leipzig EMR).

^d^ Based on 182 (H2 registry) and 2936 (Leipzig EMR) patients with valid data from cardiac imaging.

^e^ Cases transferred to another hospital were excluded.

Information on medication at hospital discharge was available for patients discharged alive with valid medication recordings (202/205, 98.5%). Overall, 87.6% of patients (177/202) received any OAC (88.0% with non‐vitamin K antagonist oral anticoagulation [NOAC], 12.0% with vitamin K antagonist [VKA]) and 80.2% of patients (162/202) were prescribed any antiplatelet. There was a lower prescription rate of OAC in the group with rPCI compared to ePCI (86.3% [151/175] vs. 96.3% [26/27], *p* < 0.001) as opposed to an increased treatment with antiplatelets (86.3% [148/175] vs. 55.2% [14/27], *p* < 0.001). When analyzing different combinations of OAC and antiplatelets, 12.4% (25/202) of all patients received no OAC (all with at least one antiplatelet), 19.8% (40/202) received any OAC without concomitant antiplatelet treatment, and 67.8% (137/202) were treated with a combination of OAC and at least one antiplatelet. Groups of rPCI and ePCI were treated differently with respect to antithrombotic drug combinations as detailed in Table 2. A majority of patients was treated with any statin (167/202, 82.7%) and/or any agent from the group or heart failure‐related drugs (197/202, 97.5%, as defined in the Supporting Information S1: Table 5) without differences with respect to the timing of PCI (*p* = 0.6 for statins, *p* > 0.9 for heart failure medication).

**TABLE 2:** Antithrombotic drug combinations within index case discharge medication.

Drug class combination	Data source							
	H2 registry^a^				Heart Center Leipzig EMR^a^			
	Overall n = 202	ePCI n = 27	rPCI n = 175	*p* value^b^	Overall n = 2963	ePCI n = 132	rPCI n = 2831	*p* value^b^
No anticoagulation with or without antiplatelet [n (%)]	25 (12.4)^c^	1 (3.7)	24 (13.7)	0.200	221 (7.5)^c^	10 (7.6)	211 (7.5)	> 0.9
Anticoagulation and no antiplatelet [n (%)]	40 (19.8)	13 (48.1)	27 (15.4)	< 0.001	191 (6.4)	61 (46.2)	130 (4.6)	< 0.001
Anticoagulation and antiplatelet [n (%)]	137 (67.8)	13 (48.1)	124 (70.9)	0.022	2,551 (86.1)	61 (46.2)	2,490 (87.9)	< 0.001

Abbreviations: EMR, Electronic medical records; ePCI, Earlier percutaneous coronary intervention performed 6–12 months before study entry; rPCI, Recent percutaneous coronary intervention performed the within index case or up to 6 months before study entry.

a Based on cases discharge alive with valid information on discharge medication.

^b^ Test for differing distributions of antithrombotic drug treatment between groups of ePCI and rPCI.

^c^ All patients of this group from the H2 registry cohort (25/25) and 92.8% from the Heart Center Leipzig EMR cohort (205/221) received at least one antiplatelet.

FU statistics were limited to patients who were discharged alive (index case) and had FU information available at 12 months (*n* = 99). Consequently, a large number of patients (*n* = 112) for whom sufficient FU data were unavailable were considered lost to FU. Events classified as major adverse events (MACE, as defined in the Supporting Information S1: Table 4) were documented in 13.7% of patients (10/73, missing data in *n* = 26). No ischemic or hemorrhagic cerebrovascular events were documented, gastrointestinal bleeding occurred in 4.0% of patients (3/75, missing data in *n* = 24), and 24.2% of patients (24/99) died within 12 months from index hospital admission. There were no associations between different patterns of antithrombotic drug combinations and any events or rehospitalizations during FU in patients with available FU information.

### 3.2 Heart Center Leipzig EMR Database

For case‐based cross‐sectional analysis, 3481 index cases met the eligibility criteria for the cross‐sectional analyses from the Heart Center Leipzig EMR database (mean age 75.8 ± 9.1 years, 27.8% female). Mean CHA_2_DS_2_‐VASc score was 5.0 ± 1.3, mean Elixhauser comorbidity score was 15.1 ± 12.2 and 81.6% (2839/3481) had an Elixhauser comorbidity index of ≥ 5. Only 235 of 3,481 patient cases (6.8%) underwent PCI between 6 and 12 months before the index case, whereas more than three quarters of patients (2712/3481, 77.9%) had documented PCI with or without coronary stent implantation during the index hospitalization. Previous or current LAAO was performed in 1.9% of patient cases (65/3481). Detailed baseline characteristics including information on distinct comorbidities are provided in Table 1.

In the EMR cohort, 2963 of 3481 cases (85.1%) were discharged alive from index hospitalization and had valid information on medication at discharge with at least one data entry. The majority of cases received any type of anticoagulant treatment (2742/2963, 92.5%) without differences between groups of cases with rPCI or ePCI (92.5% [2620/2831] vs. 92.4% [122/132], *p* > 0.9). Of them, 82.3% received a NOAC and 15.7% were treated with a VKA with a comparable distribution of OAC subgroups in rPCI and ePCI cohorts (each *p* = 0.8). Antiplatelets were prescribed in 93.0% (2756/2963) with a higher rate of prescription in the rPCI group (94.9% [2687/2831] vs. 52.3% [69/132], *p* < 0.001). Heart failure‐related drugs (2924/2963, 98.7%) and statins (2729/2963, 92.1%) were prescribed frequently without differences between the rPCI and the ePCI group (each *p* = 0.2). Grouping patient cases according to antithrombotic drug combinations at index hospital discharge, 7.5% (211/2963) had no anticoagulant treatment irrespective of existing antiplatelet therapy (of them, 92.8% or 205/221 patients were on at least one antiplatelet), 6.4% (191/2963) were prescribed an anticoagulant without concomitant antiplatelets, and 86.1% (2551/2963) received a combined antithrombotic therapy (Table 2). The prescribed drug combinations differed between the rPCI and the ePCI group (Table 2). Dual antiplatelet therapy (DAPT) at hospital discharge was documented in 17.7% of cases overall (525/2963), and a combination of any anticoagulant with DAPT was observed in 13.8% (408/2963) or 77.7% of cases with DAPT. Nontreatment with OAC was significantly more common in cases, who received LAAO or experienced acute myocardial infarction and major organ‐specific bleeding during the index hospitalization (each *p* < 0.05).

After excluding cases with documented death during index hospitalization, 2471 individual patients entered longitudinal FU analysis (per patient‐based analysis). This cohort did not differ from the overall cohort or the subgroup of patients with available medication data at index case with regard to baseline characteristics or Elixhauser comorbidity index. There were minor differences with regard to the distribution of antithrombotic drug regimens when comparing patients with medication data at baseline and those considered for FU analysis with corresponding medication data (OAC + antiplatelet therapy 86.1% vs. 87.2%; OAC without antiplatelet therapy 6.4% vs. 4.9%; *p* = 0.044). Any rehospitalization within 1 year from index hospital discharge occurred in 24.9% (615/2471), and 7.0% (172/2471) of patients were hospitalized at least twice. Hospital readmissions for any emergency reasons (cardiovascular and non‐cardiovascular) accounted for less than half of all above mentioned rehospitalizations (42.6% [262/615] of readmissions or 10.6% [262/2471] of all patients with available FU information). This includes readmissions for MACE and repeated cardiovascular revascularization (PCI or surgical revascularization) in 7.4% (184/2471) and 6.2% (154/2471) of patients. There were no readmissions for acute ischemic or hemorrhagic stroke, and readmissions for major organ specific bleeding were rare (4/2471, 0.2%). In multivariable analyses (including baseline characteristics, PCI timing, drug treatment), the prescription of anticoagulants without concomitant antiplatelet treatment was associated with an increased risk for rehospitalizations for repeated coronary revascularization (OR 2.26, 95% CI 1.17–4.37, *p* = 0.02), and MACE (OR 2.29, 95% CI 1.24–4.22, *p* = 0.01). No other predictors were identified for various FU endpoints.

### 3.3 Patterns of Antithrombotic Drug Treatment

Comparing baseline characteristics of patients within the H2 registry and the Heart Center Leipzig EMR database stratified for different antithrombotic drug combinations, relevant differences were observed (Tables 3a and 3b). Additional analyses of baseline characteristics, which stratify patient cases for the treatment with anticoagulants overall, are provided in the supplementary material (Supporting Information S1: Tables 6a and 6b). In the H2 registry cohort, the prevalence of comorbidities did not differ between groups except for documented end‐stage renal dysfunction with hemodialysis. In the larger Heart Center Leipzig EMR cohort, patients being not prescribed with any anticoagulant had more frequently chronic kidney disease, peripheral vascular disease, coagulopathy, fluid and electrolyte disorders, and blood loss anemia (each *p* < 0.05). In multivariable analysis, higher age was associated with an increased utilization of anticoagulants (Heart Center Leipzig EMR cohort), whereas higher comorbidity burden based on the Elixhauser comorbidity score (Heart Center Leipzig EMR cohort), the treatment with an antiplatelet (H2 registry cohort), and a prior LAAO procedure (both cohorts) were associated with lower prescription rates of anticoagulants (Table 4).

**TABLE 3a:** Baseline characteristics stratified for antithrombotic drug combinations (H2 registry).

Variable	AC and AP	AC and no AP	No AC ± AP	*p value* a
Mean age [years]	75.2 ± 8.6	74.8 ± 8.5	71.7 ± 11.2	0.200
Sex, female [n (%)]	41 (29.9)	17 (42.5)	3 (12.0)	0.200
CHA_2_DS_2_‐VASc score	5.5 ± 1.3	5.6 ± 1.5	5.0 ± 1.4	0.200
Dialysis [n (%)]	3 (2.2)	2 (5.0)	5 (20.0)	< 0.001
LAAO [n (%)]	3 (2.2)	0 (0.0)	6 (25.0)	< 0.001

*Note:* Patient characteristics are based on cases with available medication data.

Abbreviations: AC, Anticoagulant; AT, Antiplatelet; LAAO, Left atrial appendage occlusion.

a One‐way ANOVA/Pearson's Chi‐squared test.

**TABLE 3b:** Baseline characteristics stratified for antithrombotic drug combinations (Heart Center Leipzig EMR database).

Variable	AC and AP	AC and no AP	No AC ± AP	*p value* a
Mean age [years]	76.3 ± 8.6	74.0 ± 9.3	74.4 ± 11.3	< 0.001
Sex, female [n (%)]	852 (28.1)	49 (23.7)	66 (28.2)	0.4
CHA_2_DS_2_‐VASc score	5.0 ± 1.2	4.9 ± 1.3	4.9 ± 1.4	0.2
Elixhauser comorbidity score	13.4 ± 11.0	15.2 ± 13.0	18.0 ± 13.1	< 0.001
LAAO [n (%)]	36 (1.2)	5 (2.4)	24 (10.3)	< 0.001

*Note:* Patient characteristics are based on cases with available medication data.

Abbreviations: AC, Anticoagulant; AP, Antiplatelet; LAAO, Left atrial appendage occlusion.

a One‐way ANOVA/Pearson's Chi‐squared test.

**TABLE 4:** Multivariable analysis for the treatment with anticoagulants.

Variable	Data source			
	H2 registry		Heart Center Leipzig EMR	
	Odds ratio (95% CI)	*p value*	Odds ratio (95% CI)	*p value*
Sex, male	0.424 (0.09–2.06)	0.288	1.27 (0.88–1.83)	0.198
Age	1.61 (0.91–2.85)	0.104	1.21 (1.02–1.43)	0.032
CHA_2_DS_2_‐VASc score	2.17 (0.12–38.5)	0.599	1.96 (0.83–4.61)	0.125
Elixhauser comorbidity score	/	/	0.55 (0.45–0.68)	< 0.001
LAAO	0.02 (0.01–0.14)	< 0.001	0.11 (0.06–0.20)	< 0.001
rPCI versus ePCI	0.25 (0.02–3.20)	0.286	0.78 (0.37–1.64)	0.516
Treatment with antiplatelets	0.0 (0.0–0.48)	0.002^a^	0.87 (0.49–1.57)	0.652

Abbreviations: CI, Confidence interval; ePCI, Earlier percutaneous coronary intervention performed 6–12 months before study entry; LAAO, Left atrial appendage occlusion; rPCI, Recent percutaneous coronary intervention performed the within index case or up to 6 months before study entry.

a Based on Fisher‐Test because there were no patients without both AC anticoagulants and antiplatelets.

## 4 Discussion

With this study, we provide insight into the current real‐world medical management of patients with coexisting AF and CAD in Germany. OAC prescription rates were high, but there was still a need for further improvement to be seen. We were able to show that certain drug regimens were associated with clinical events during FU.

In previous larger studies examining OAC treatment in patients with AF in general, prescription rates were consistent with the results of our analyses. Data from the GARFIELD‐AF registry, collected between 2012 and 2016, showed an overall rate of OAC treatment of 80.1% in patients with newly diagnosed nonvalvular AF with at least one risk factor for thromboembolic events, which increased to 84.3% in the subgroup of patients with a CHA_2_DS_2_‐VASc score of ≥ 2 points [29]. Of the remaining patients, 10.7% were prescribed an antiplatelet and 9.3% did not receive any antithrombotic therapy [29]. In comparison, the vast majority of patients not on OACs were on antiplatelet therapy in both cohorts evaluated in our study. In the EurObservational Research Programme in Atrial Fibrillation (EORP‐AF) long‐term registry, 86.9% of patients were reported to be treated with any OAC (mean CHA_2_DS_2_‐VASc score 3.3 and 2.9 points in OAC groups), while 7.0% were prescribed antiplatelet therapy alone (despite a mean CHA_2_DS_2_‐VASc score of 3.4 points in this subgroup), and 6.1% received no antithrombotic therapy (mean CHA_2_DS_2_‐VASc score 1.9 points) [30]. Consistent with previous studies, OAC treatment was associated with a lower risk of any thromboembolic event, acute coronary syndrome, or cardiovascular death during FU [30]. In other reports from the EORP‐AF long‐term registry, the proportion of patients receiving OAC therapy was comparable to the H2 registry cohort of our study, but lower compared to the Heart Center Leipzig EMR cohort [31, 32]. Possible reasons for the higher prescription rate in the latter cohort may be the higher average thromboembolic risk (compared to EORP‐AF with a mean CHA_2_DS_2_‐VASc score of 3.1 points) as well as the data source itself (single‐center database of a high‐volume specialized cardiovascular center compared to multicenter databases with different types of center specification). Recent administrative database analyses that included AF cases early after initial medical contact provided evidence of significant underuse of OAC in real‐world cohorts, which was associated with poorer outcomes in patients without OAC [33, 34]. Only 61.1% of patients in an Italian cohort were treated with OAC, compared to 52.2% of patients considered at high risk for thromboembolism risk in an US database (mean CHA_2_DS_2_‐VASc score 4.7 points) [33, 34]. Neither of the data sources used in our study determines the time of AF diagnosis, making it difficult to directly compare cohorts in this regard.

Several factors have been identified that are associated with lower quality of medical care for AF. Ding and colleagues reported lower prescription rates and more underdosing of OAC in patients with chronic kidney disease, a comorbidity diagnosed in more than half of the patients in our study [31]. There are conflicting data regarding the effect of age on OAC prescription rates, with studies supporting both higher and lower prescribing in the elderly [12, 30, 35, 36]. Other factors that have been associated with fewer OAC prescriptions in previous studies include frailty, higher comorbidity burden in general, comorbid CAD, concomitant cancer, and a higher bleeding risk, including concomitant antithrombotic and especially antiplatelet therapy, which is largely consistent with our results [12, 35, 36, 37, 38, 39]. Bleeding risk can only be estimated in our dataset, as not all components of relevant assessment tools such as the HAS‐BLED score or the ABC score are available in our data. However, it is assumed that the mean HAS‐BLED score would indicate an increased bleeding risk (≥ 3 points) due to the high mean age in our cohort, the relevant comorbidity burden, and the high proportion of patients on antiplatelet therapy. The high 12‐month mortality rate in our analysis (H2) also indicates a high‐risk patient population, comparable to other studies examining cohorts with concomitant heart failure, AF and CAD [40].

Focusing on analyses of patients with coexisting AF and CAD undergoing recent PCI, the proportion of patients receiving OAC therapy varied widely, ranging from less than 50% to nearly 100% in different studies [41, 42, 43, 44, 45]. Over time, more patients received dual or triple antithrombotic therapy rather than DAPT alone, consistent with guideline recommendations [45, 46, 47]. Comorbidity burden and a history of stroke were important predictors of OAC treatment, whereas concomitant antiplatelet therapy was associated with non‐use of OACs [12, 14, 45, 46]. Treatment with OACs, and particularly NOACs, was associated with a net clinical benefit in terms of both MACE and major bleeding events in patients with AF and CAD in general [48, 49]. However, other analyses focusing on patients with recent PCI have reported only trends toward improved outcomes in patients on OAC, which is consistent with our findings.

There are clear recommendations from European guidelines and consensus statements regarding the optimal drug regimens and duration of combined antithrombotic therapy in patients with AF and CAD who have recently undergone coronary revascularization [7, 19, 20, 21, 22, 23]. At hospital discharge, a combination of an OAC and an antiplatelet drug for 6 and 12 months after revascularization for chronic coronary syndrome and acute coronary syndrome is the standard recommendation for most patients followed by long‐term OAC treatment alone. In our analysis, even in the rPCI groups, 15.2% (H2 registry) and 5.1% (Heart Center Leipzig EMR cohort) were discharged without antiplatelet therapy, reflecting the decision to shorten the duration of combined antithrombotic therapy. Specific reasons at the patient level cannot be derived from our analysis, but a lack of antiplatelet treatment was associated with increased MACE rates during FU. On the other hand, 14.5% (H2 registry) and 7.5% (Heart Center Leipzig EMR cohort) of patients were not prescribed OAC therapy at hospital discharge with the vast majority of these patients being treated with one or more antiplatelet agents. As OAC may be beneficial even in patients at high risk of bleeding, this must be considered a potential underuse [50]. Importantly, previous studies have shown insufficient agreement between physician assessment of bleeding risk and validated scores, highlighting the need for standardized risk assessment [42].

## 5 Limitations

The study is based on inpatient data from hospitals of a private German health care provider; therefore, the generalizability of our data to the entire German or other health care systems can be discussed. When interpreting the results of the FU analysis, it is important to consider that only cases hospitalized in the index hospital were considered in the Heart Center Leipzig EMR cohort and patients with acute cerebrovascular events may be primarily admitted to different hospitals with neurological specialization. On the other hand, there was a large number of patients from the H2 registry had insufficient or incomplete FU datasets, resulting in 53.1% of patients being considered as lost to FU.

This study is based in part on administrative data that were collected for remuneration rather than research purposes, which could potentially influence the encoded information and harbors a risk of bias in the assessment of various clinical endpoints [51]. The quality of results is highly dependent on the correct coding of procedures and diagnoses at hospital discharge [52]. However, there is ongoing evaluation of discharge diagnoses, appropriateness of hospitalization, and coding by health insurances, which supports the assumption of overall valid information. This includes the information presented in this study as it is relevant for reimbursement. It has been shown, that coding accuracy of ICD‐10‐GM‐based first discharge diagnoses and OPS codes in administrative data is good and reliable [53, 54]. However, there may be a nonquantifiable risk of underreporting associated with the calculation of the CHADS‐VASc score based on administrative data.

There is a proportion of patient cases without valid discharge medication information that were excluded from the analyses presented, with a corresponding risk of bias in the results for overall drug treatment. The H2 registry data did not differentiate between single and dual antiplatelet therapy because this was not the original purpose of the study. Lastly, we did not evaluate the appropriateness of OAC dosing, which is a common issue in patients with CAD [35, 55, 56].

## 6 Conclusion

The present study presents real‐world data on antithrombotic drug therapy in patients with AF and coexisting CAD, who underwent PCI within the preceding year. We reported patient numbers treated with different regimens of antithrombotic therapy and identified several factors like comorbidity burden and concomitant antiplatelet treatment that were associated with their non‐prescription. Our results can serve as a data basis for further quality improvement initiatives in this relevant patient population.

## Ethics Statement

This study has been approved by the local ethics committee of the University of Leipzig and the Helios Kliniken GmbH data protection authority. The corresponding author affirms that this manuscript is an honest, accurate and transparent account of the study being reported; that no important aspects of the study have been omitted; and that any discrepancies from the study as planned have been explained. There has been no public involvement associated with this study.

## Conflicts of Interest

All authors state that there is nothing to declare. All authors have completed the ICMJE uniform disclosure form (at http://www.icmje.org/disclosure-of-interest/) and declare: no support from any organization for the submitted work; no financial relationship to Pfizer Pharma GmbH; no financial relationships with any organizations that might have an interest in the submitted work in the previous 3 years; no other relationships or activities that could appear to have influenced the submitted work.

## Supporting information


**Supplemental Table 1:** ICD‐10 and OPS codes used to identify eligible patient cases.
**Supplemental Table 2:** ICD‐codes used to calculate Elixhauser comorbidity index.
**Supplemental Table 3:** ICD‐codes used to calculate CHA2DS2‐VASc‐Score.
**Supplemental Table 4:** Variable definition for in‐hospital treatments and outcomes at readmission.
**Supplemental Table 5:** Medication of interest based on ATC codes.
**Supplemental Table 6a:** Baseline characteristics stratified for the treatment with anticoagulants (H2 registry).
**Supplemental Table 6b:** Baseline characteristics stratified for the treatment with anticoagulants (Heart Center Leipzig EMR database).

## Data Availability

The data and computing codes underlying this article will be shared on reasonable request to the corresponding author.
