# Supplementary material for: Management of Antithrombotic Therapy in Patients With Coexisting Atrial Fibrillation and Coronary Artery Disease Who Underwent Percutaneous Coronary Intervention Within the Last Year
Source: Clin Cardiol. 2025 Oct 16;48(10):e70196. doi: 10.1002/clc.70196 (PMC12529229; doi:10.1002/clc.70196)
Supplement: Supplementary file 1 — Supplemental Table 1: ICD‐10 and OPS codes used to identify eligible patient cases. Supplemental Table 2: ICD‐codes used to calculate Elixhauser comorbidity index. Supplemental Table 3: ICD‐codes used to calculate CHA2DS2‐VASc‐Score. Supplemental Table 4: Variable definition for in‐hospital treatments and outcomes at readmission. Supplemental Table 5: Medication of interest based on ATC codes. Supplemental Table 6a: Baseline characteristics stratified for the treatment with anticoagulants (H2 registry). Supplemental Table 6b: Baseline characteristics stratified for the treatment with anticoagulants (Heart Center Leipzig EMR database). [file CLC-48-e70196-s001.docx]

**Supplemental Material**

Supplemental Table 1: ICD-10 and OPS codes used to identify eligible patient cases

Supplemental Table 2: ICD-codes used to calculate Elixhauser comorbidity index (weighting according to AHRQ algorithm)

Supplemental Table 3: ICD-codes used to calculate CHA_2_DS_2_-VASc-Score

Supplemental Table 4: Variable definition for in-hospital treatments and outcomes at readmission

Supplemental Table 5: Medication of interest based on ATC codes

Supplemental Table 6: Baseline characteristics stratified for the treatment with anticoagulants

**Supplemental Table 1: ICD-10 and OPS codes used to identify eligible patient cases**

| ICD-10 and OPS codes used to identify eligible patient cases | | |
| --- | --- | --- |
| *Condition* | *ICD-10 codes* | *OPS codes* |
| AF / atypical atrial flutter | I48.0, I48.1, I48.2, I48.4 | / |
| CAD | I21, I22, I23.0, I23.1, I23.2, I23.3, I23.4, I23.5, I23.6, I23.8, I25.0, I25.11, I25.12, I25.13, I25.14, I25.15, I25.16, I25.17, I25.18, I25.19, I25.2, I25.3, I25.4, I25.5, I25.6, I25.8, I25.9 | / |
| PCI | / | 8-837.0, 8-837.1, 8-837.2, 8-837.5, 8-837.6, 8-837.8, 8-837.9, 8-837.k, 8-837.m, 8-837.p, 8-837.q, 8-837.t, 8-837.u, 8-837.v, 8-837.w, 8-839.9, 8-83d.0, 8-83d.1, 8-83d.2, 8-83d.6 |

AF: Atrial fibrillation; CAD: Coronary artery disease; ICD-10: International Statistical Classification of Diseases and Related Health Problems (German Modification); OPS: Operations and Procedures; PCI: Percutaneous coronary intervention

**Supplemental Table 2: ICD-codes used to calculate Elixhauser comorbidity index**

| ICD-10 codes used to calculate Elixhauser comorbidity index | | |
| --- | --- | --- |
| *Item* | *Weight* | *ICD-10 codes* |
| AIDS / HIV | 0 | B20, B21, B22, B23, B24 |
| Alcohol Abuse | -1 | F10, E52, G62.1, I42.6, K29.2, K70.0, K70.3, K70.9, T51, Z50.2, Z71.4, Z72.1 |
| Blood Loss Anemia | -3 | D50.0 |
| Cardiac Arrhythmias | 0 | I44.1, I44.2, I44.3, I45.6, I47, I48, I49, R00.0, R00.1, R00.8, T82.1, Z45.00, Z45.01, Z95.0 |
| Chronic Pulmonary Disease | 3 | I27.8, I27.9, J40, J41, J42, J43, J44, J45, J46, J47, J60, J61, J62, J63, J64, J65, J66, J67, J68.4, J70.1, J70.3 |
| Chronic Renal Failure | 6 | I12.0, I31.1, N18, N19, N25.0, Z49.0, Z49.1, Z49.2, Z94.0, Z99.2 |
| Coagulopathy | 11 | D65, D66, D67, D68, D69.1, D69.3, D69.4, D69.5, D69.6 |
| Congestive Heart Failure | 9 | I09.0, I11.0, I13.0, I13.2, I25.5, I42.0, I42.1, I42.2, I42.5, I42.6, I42.7, I42.8, I42.9, I43, I50 |
| Deficiency Anemia | -2 | D50.8, D50.9, D51, D52, D53 |
| Depression | -5 | F20.4, F31.3 - F31.5, F32, F33, F34.1, F41.2, F43.2 |
| Diabetes Mellitus, Uncomplicated | 0 | E10.0, E10.1, E10.9, E11.0, E11.1, E11.9, E12.0, E12.1, E12.9, E13.0, E13.1, E13.9, E14.0, E14.1, E14.9 (excluding E10.2, E10.3, E10.4, E10.5, E10.6, E10.7, E10.8, E11.2, E11.3, E11.4, E11.5, E11.6, E11.7, E11.8, E12.2, E12.3, E12.4, E12.5, E12.6, E12.7, E12.8, E13.2, E13.3, E13.4, E13.5, E13.6, E13.7, E13.8, E14.2, E14.3, E14.4, E14.5, E14.6, E14.7, E14.8) |
| Diabetes Mellitus, Complicated | -3 | E10.2, E10.3, E10.4, E10.5, E10.6, E10.7, E10.8, E11.2, E11.3, E11.4, E11.5, E11.6, E11.7, E11.8, E12.2, E12.3, E12.4, E12.5, E12.6, E12.7, E12.8, E13.2, E13.3, E13.4, E13.5, E13.6, E13.7, E13.8, E14.2, E14.3, E14.4, E14.5, E14.6, E14.7, E14.8 |
| Drug Abuse | -7 | F11, F12, F13, F14, F15, F16, F18, F19, Z71.5, Z72.2 |
| Fluid And Electrolyte Disorders | 11 | E22.2, E86, E87 |
| Hypertension (combined uncomplicated and complicated) | -1 | I10, I11, I12, I13, I15 |
| Hypothyroidism | 0 | E00, E01, E02, E03, E89.0 |
| Liver Disease | 4 | B18, I85, I86.4, I98.2, K70, K71.1, K71.3, K71.4, K71.5, K71.7, K72, K73, K74, K76.0, K76.2, K76.9, Z94.4 |
| Lymphoma | 6 | C81, C82, C83, C84, C85, C88, C96, C90.0, C90.2 |
| Metastatic Cancer | 14 | C77, C78, C79, C80 |
| Neurological Disorders, other | 5 | G10, G11, G12, G13. G20, G21, G22, G25.4, G25.5, G31.2, G31.8, G31.9, G32, G35, G36, G37, G40, G41, G93.1, G93.4, R47.0, R56 |
| Obesity | -5 | E66 |
| Paralysis | 5 | G04.1, G11.4, G80.1, G80.2, G81, G82, G83.0, G83.1, G83.2, G83.3, G83.4, G83.9 |
| Peptic Ulcer Disease, Excluding Bleeding | 0 | K25.7, K25.9, K26.7, K26.9, K27.7, K27.9, K28.7, K28.9 |
| Peripheral Vascular Disorders | 3 | I70, I71, I73.1, I73.8, I73.9, I77.1, I79.0, I79.2, Z95.81, Z95.88, Z95.9 |
| Psychoses | -5 | F20, F22, F23, F24, F25, F28, F29, F30.2, F31.2, F31.5 |
| Pulmonary Circulation Disorder | 6 | I26, I27, I28.0, I28.8, I28.9 |
| Rheumatoid Arthritis / Collagen Vascular Diseases | 0 | L94.0, L94.1, L94.3, M05, M06, M08, M12.0, M12.3, M30, M31.0, M31.1, M31.2, M31.3, M32, M33, M34, M35, M45, M46.1, M46.8, M46.9 |
| Solid Tumor Without Metastases | 7 | C00, C01, C02, C03, C04, C05, C06, C07, C08, C09, C10, C11, C12, C13, C14, C15, C16, C17, C18, C19, C20, C21, C22, C23, C24, C25, C26, C30, C31, C32, C33, C34, C37, C38, C39, C40, C41, C43, C45, C46, C47, C48, C49, C50, C51, C52, C53, C54, C55, C56, C57, C58, C60, C61, C62, C63, C64, C65, C66, C67, C68, C69, C70, C71, C72, C73, C74, C75, C76, C97 |
| Valvular Heart Disease | 0 | I05, I06, I07, I08, I09.1, I34, I35, I36, I37, I38, I39, Q23.0, Q23.1, Q23.2, Q23.3, Z95.2, Z95.3, Z95.4 |
| Weight Loss | 9 | E40, E41, E42, E43, E44, E45, E46, R63.4, R64 |

ICD-10: International Statistical Classification of Diseases and Related Health Problems (German Modification)

**Supplemental Table 3: ICD-codes used to calculate CHA_2_DS_2_-VASc-Score**

| ICD-10 codes used to calculate CHA_2_DS_2_-VAsc-Score | | |
| --- | --- | --- |
| *Condition* | *Weight* | *ICD-10 codes* |
| Congestive heart failure | 1 | I11.0, I13.0, I13.2, I25.5, I42.0, I42.1, I42.2, I42.5, I42.6, I42.7, I42.8, I42.9, I43.0, I43.1, I43.2, I43.8, I50.0, I50.1, I50.9 |
| Hypertension | 1 | I10.x, I11.x, I12.x, I13.x, I15.x, I67.4 |
| Age ≥75 years | 2 | N/A |
| Diabetes mellitus | 1 | E10.x, E11.x, E12.x, E13.x, E14.x, E15 |
| Previous stroke / TIA | 2 | I61.x, I63.x, I64.x, I69.x |
| Vascular disease | 1 | I25.x, I65.x, I66.x, I67.9, I70.x, I74.x |
| Age 65-74 years | 1 | N/A |
| Sex category (female) | 1 | N/A |

ICD-10: International Statistical Classification of Diseases and Related Health Problems (German Modification)

All ICD-10-GM codes ending on “.x” are meant as coding groups that include all corresponding sub-codes.

**Supplemental Table 4: Variable definition for in-hospital treatments and outcomes at readmission**

| ICD-10 codes used to define in-hospital treatments of interest and events for longitudinal analysis | | |
| --- | --- | --- |
| *Condition* | *ICD-10 codes* | *OPS codes* |
| Myocardial infarction | I21, I22, I25.2 | / |
| Coronary revascularization | / | 5-361, 5-362, 5-363, 5-364, 8-837.3 8-837.k, 8-837.m, 8-837.n, 8-837.p, 8-837.u, 8-837.v, 8-837.w, 8-837.0, 8-837.1, 8-837.2, 8-837.5, 8-837.6, 8-837.q, 8-837.t |
| CABG | / | 5-361, 5-362, 5-363, 5-364 |
| PCI with stent | / | 8-837.3, 8-837.k, 8-837.m, 8-837.n, 8-837.p, 8-837.u, 8-837.v, 8-837.w, 8-839.9, 8-83d.0, 8-83d.1, 8-83d.2 |
| PCI without stent* | / | 8-837.0, 8-837.1, 8-837.2, 8-837.5, 8-837.6, 8-837.q, 8-837.t, 8-83d.6 |
| LAAO | / | 5-379.1, 8-837.s0, 8-837.s1, 8-837.sx |
| Unstable angina | I20.0 | / |
| Angina pectoris | I20.1, I20.8, I20.9 | / |
| Stroke | G45, I61, I63, I64, I69.1, I69.2, I69.3, I69.4, I69.8 | / |
| Major organ specific bleeding | D62, I60, I61, I62, I85.0, K22.6, K25.0, K25.2, K25.4, K25.6, K26.0, K26.2, K26.4, K26.6, K27.0, K27.2, K27.4, K27.6, K28.0, K28.2, K28.4, K28.6, K29.0, K62.5, K92.0, K92.1, K92.2 | / |
| MACE | I21, I22, I25.2, I20.0, I20.1, I20.8, I20.9, G45, I61, I63, I64, I69.1, I69.2, I69.3, I69.4, I69.8, D62, I60, I61, I62, I85.0, K22.6, K25.0, K25.2, K25.4, K25.6, K26.0, K26.2, K26.4, K26.6, K27.0, K27.2, K27.4, K27.6, K28.0, K28.2, K28.4, K28.6, K29.0, K62.5, K92.0, K92.1, K92.2 | 5-361, 5-362, 5-363, 5-364, 8-837.3 8-837.k, 8-837.m, 8-837.n, 8-837.p, 8-837.u, 8-837.v, 8-837.w, 8-839.9, 8-83d.0, 8-83d.1, 8-83d.2, 8-837.0, 8-837.1, 8-837.2, 8-837.5, 8-837.6, 8-837.q, 8-837.t, 8-83d.6 |
| Cardiovascular death^#^ | Any ICD-10 diagnoses from the I group |  |

CABG: Coronary artery bypass grafting; ICD-10: International Statistical Classification of Diseases and Related Health Problems (German Modification); LAAO: Left atrial appendage occlusion; MACE: Major adverse cardiovascular event; OPS: Operations and Procedures; PCI: Percutaneous coronary intervention

* Excluding cases with following encoded OPS codes (these cases are only counted in the group PCI with stent): 8-837.3, 8-837.k, 8-837.m, 8-837.n, 8-837.p, 8-837.u, 8-837.v, 8-837.w, 8-839.9, 8-83d.0, 8-83d.1, 8-83d.2

# Excluding cases with following ICD-10 codes as a main diagnosis at hospital discharge: I89.0, I97.2, I97.8, I97.80, I97.81, I97.82, I97.83, I97.84, I97.85, I97.86, I97.87, I97.88

For in-hospital treatments during the index case, both main and secondary diagnoses were considered (except for the variable “cardiovascular death” that was based on main diagnoses only). For readmission statistics, only main diagnoses from readmission cases were considered relevant.

**Supplemental Table 5: Medication of interest based on ATC codes**

| ATC codes used to define medication of interest | | |
| --- | --- | --- |
| *Medication group* | *Drug class* | *ATC codes* |
| Anticoagulants | Novel oral anticoagulants | B01AE07, B01AF01, B01AF02, B01AF03 |
|  | Vitamin K antagonists | B01AA03, B01AA04 |
|  | Heparins | B01AB01, B01AB04, B01AB05, B01AB06, B01AB07, B01AB08, B01AB09, B01AB10, B01AB13, B01AB51, B01AB63 |
| Antiplatelets | Low-dose aspirin | B01AC06 |
|  | P2Y12 inhibitors | B01AC04, B01AC05, B01AC22, B01AC24, B01AC25 |
|  | Other antiplatelets | B01AC07, B01AC09, B01AC11, B01AC13, B01AC16, B01AC17, B01AC21 |
| Heart failure-relevant drugs | ACE inhibitors | C09A, C09B, C10BX04, C10BX06, C10BX07, C10BX11, C10BX12, C10BX13, C10BX14, C10BX15, C10BX17, C10BX18 |
|  | Angiotensin receptor blockers | C09C, C09DA, C09DB, C09DX01, C09DX02, C09DX03, C09DX05, C09DX06, C09DX07, C09DX08, C09DX09, C10BX10, C10BX16 |
|  | Renin inhibitors | C09XA02, C09XA52, C09XA53, C09XA54 |
|  | ARNI | C09DX04 |
|  | Beta receptor blockers | C07, C09BX02, C09BX04, C09BX05, C09DX05 |
|  | High ceiling diuretics | C03C, C03EB, C03ED, C03ED01, C07CA51, C07CA52, C09BA55 |
|  | MRA | C03DA, C03EC, C03ED |
| Diabetes-relevant drugs | Metformin | A10BA, A10BD |
|  | Sulfonylureas | A10BB |
|  | DPP4 inhibitors | A10BH, A10BD07, A10BD08, A10BD10 |
|  | SGLT2 inhibitors | A10BK01, A10BK02, A10BK03, A10BD15, A10BD16, A10BD20 |
|  | GLP1 agonists | A10BJ |
|  | Metiglinides | A10BX02, A10BX03, A10BD03, A10BD04, A10BD05 |
|  | Glitazones | A10BG |
|  | Acarbose | A10BF |
|  | Insulins | A10AB, A10AC, A10AD, A10AE |
| Risk factor management | Statins | C10AA, C10BA, C10BX |

ACE: Angiotensin converting enzyme; ARNI: Angiotensin receptor blockers + neprilysin inhibitors; ATC: Anatomical therapeutic chemical; DPP4: Dipeptidyl peptidase-4; GLP1: Glucagon-like peptide 1; MRA: mineralocorticoid receptor antagonists; SGLT2: Sodium glucose cotransporter type 2

**Supplemental Table 6a: Baseline characteristics stratified for the treatment with anticoagulants (H2 registry)**

| Variable | Anticoagulation* | No anticoagulation* | p value^†^ |
| --- | --- | --- | --- |
|  | n=177 | n=25 |  |
| Mean age [years] | 75.1±8.5 | 71.7±11.2 | 0.070 |
| Sex, female [n/N (%)] | 58/177 (32.8) | 3/25 (12.0) | 0.059 |
| BMI [kg/m²] | 28.6±5.6 | 27.7±4.7 | 0.400 |
| CHA_2_DS_2_-VASc score | 5.5±1.4 | 5.0±1.4 | 0.053 |
| LVEF^‡^ [%] | 41.8±13.7 | 43.6±13.8 | 0.600 |
| eGFR [ml/min/1.73m²] | 53.3±21.7 | 52.2±34.2 | 0.800 |
| PCI within index case or up to 6 months prior to study entry [n/N (%)] | 151/177 (85.3) | 24/25 (96.0) | 0.200 |
| LAAO [n/N (%)] | 3/176 (1.7) | 6/24 (25.0) | <0.001 |
| Hypertension [n/N (%)] | 23/25 (92.0) | 165/177 (93.2) | >0.9 |
| Heart failure [n/N (%)] | 177/177 (100.0) | 25/25 (100.0 | >0.9 |
| HFrEF based on LVEF [n/N (%)] | 68/157 (43.3) | 10/23 (43.5) | >0.9 |
| CKD [n/N (%)] | 104/176 (59.1) | 15/25 (60.0) | >0.9 |
| Diabetes mellitus [n/N (%)] | 84/176 (47.7) | 10/25 (40.0) | 0.600 |
| PVD [n/N (%)] | 59/176 (33.5) | 7/25 (28.0) | 0.700 |
| Cerebrovascular disease [n/N (%)] | 32/176 (18.2) | 3/25 (12.0) | >0.9 |
| Gastrointestinal bleeding [n/N (%)] | 9/176 (5.1) | 3/25 (12.0) | 0.400 |
| Chronic pulmonary disease [n/N (%)] | 25/175 (14.3) | 6/25 (24.0) | 0.300 |
| In-hospital mortality at index case [n/N (%)] | 0/177 (0.0) | 0/25 (0.0) | >0.9 |

BMI: Body mass index; CKD: Chronic kidney disease; eGFR: Estimated glomerular filtration rate; HFrEF: Heart failure with reduced ejection fraction; LAAO: Left atrial appendage occlusion; PVD: Peripheral vascular disease

Patient characteristics are based on cases with available medication data; n/N refers to available data for the specific variable

* Presented as means with standard deviation or proportions

† One-way ANOVA / Pearson’s Chi-squared test

‡ Missing data for n=30 patients (n=26 + n=4 per group)

**Supplemental Table 6b: Baseline characteristics stratified for the treatment with anticoagulants (Heart Center Leipzig EMR database)**

| Variable | Anticoagulation* | No anticoagulation* | p value^†^ |
| --- | --- | --- | --- |
|  | n=2.795 | n=234 |  |
| Mean age [years] | 76.1±8.7 | 74.4±11.3 | 0.003 |
| Sex, female [n/N (%)] | 776 (27.8) | 66/234 (28.2) | >0.9 |
| BMI [kg/m²] | 28.4±5.0 | 27.9±5.5 | 0.200 |
| CHA_2_DS_2_-VASc score | 5.0±1.2 | 4.9±1.4 | 0.200 |
| Elixhauser comorbidity score | 13.6±11.2 | 18.0±13.1 | <0.001 |
| LVEF^‡^ [%] | 47.0±14.0 | 45.4±14.9 | 0.100 |
| eGFR [ml/min/1.73m²]^§^ | 58.3±21.8 | 54.2±29.5 | 0.008 |
| PCI within index case or up to 6 months prior to study entry [n/N (%)] | 2,672/2,795 (95.6) | 224/234 (95.7) | >0.9 |
| LAAO [n/N (%)] | 32/2,795 (1.1) | 24/234 (10.3) | <0.001 |
| Hypertension [n/N (%)] | 2,583/2,795 (92.4) | 206/234 (88.0) | 0.200 |
| Heart failure [n/N (%)] | 2,009/2,795 (71.9) | 174/234 (74.4) | 0.500 |
| HFrEF based on LVEF [n/N (%)] | 773/2,328 (33.2) | 80/212 (37.7) | 0.100 |
| Valvular heart disease [n/N (%)] | 1,097/2,795 (39.2) | 112/234 (47.9) | 0.012 |
| CKD [n/N (%)] | 1,538/2,795 (55.0) | 150/234 (64.1) | 0.009 |
| Diabetes mellitus [n/N (%)] | 1,230/2,795 (44.0) | 110/234 (47.0) | 0.400 |
| PVD [n/N (%)] | 1,138/2,795 (40.7) | 125/234 (53.4) | <0.001 |
| Cerebrovascular disease [n/N (%)] | 85/2,795 (3.0) | 9/234 (3.8) | 0.500 |
| Coagulopathy [n/N (%)] | 177/2,795 (6.3) | 35/234 (15.0) | <0.001 |
| Blood loss anemia [n/N (%)] | 6/2,795 (0.2) | 3/234 (1.3) | 0.024 |
| Chronic pulmonary disease [n/N (%)] | 252/2,795 (9.0) | 29/234 (12.4) | 0.110 |
| In-hospital mortality at index case [n/N (%)] | 53/2,795 (1.9) | 13/234 (7.7) | <0.001 |

BMI: Body mass index; CKD: Chronic kidney disease; eGFR: Estimated glomerular filtration rate; HFrEF: Heart failure with reduced ejection fraction; LAAO: Left atrial appendage occlusion; PVD: Peripheral vascular disease

Patient characteristics are based on cases with available medication data (but not to those discharged alive explaining the differing n to the data presented in the main manuscript); n/N refers to available data for the specific variable

* Presented as means with standard deviation or proportions

† One-way ANOVA / Pearson’s Chi-squared test

‡ Missing data for n=553 patients (n=525 + n=28 per group)

§ Missing data for n=22 patients (n=20 + n=2 per group)
